# Comprehensive evaluation of military training-induced fatigue among soldiers in China: A Delphi consensus study

**DOI:** 10.3389/fpubh.2022.1004910

**Published:** 2022-11-29

**Authors:** Yi Ruan, Shang-jin Song, Zi-fei Yin, Man Wang, Nian Huang, Wei Gu, Chang-quan Ling

**Affiliations:** ^1^Faculty of Traditional Chinese Medicine, Naval Medical University, Shanghai, China; ^2^PLA Naval Medical Center, Shanghai, China; ^3^Department of Traditional Chinese Medicine, Xingcheng Sanatorium of PLA Strategic Support Force, Xingcheng, China

**Keywords:** evaluation, military training, fatigue, soldier, Delphi study

## Abstract

**Objective:**

Military training-induced fatigue (MIF) often results into non-combat attrition. However, standard evaluation of MIF is unavailable. This study aimed to provide credible suggestions about MIF-evaluation.

**Methods:**

A 3-round Delphi study was performed. The authority of the experts was assessed by the authority coefficient (*Aa*). In round 1, categories of indicators were collected *via* anonymous survey of experts, then potential indicators were selected *via* literature search. In round 2, experts should evaluate the clinical implication, practical value, and importance of each potential indicators, or recommend new indicators based on feedback of round 1. Indicators with recommendation proportions ≥ 70% and new recommended indicators would be included in round 3 to be rated on a 5-point Likert scale. “Consensus in” was achieved when coefficient of concordance (Kendall's *W*) of a round was between 0.2 and 0.5 and the coefficient of variation (*CV*) of each aspect for an indicator was < 0.5. If round 3 could not achieve “consensus in,” more rounds would be conducted iteratively based on round 3. Indicators included in the recommendation set were ultimately classified into grade I (highly recommended) or grade II (recommended) according to the mean score and *CV* of the aspects.

**Results:**

Twenty-three experts participated with credible authority coefficient (mean *Aa* = 0.733). “Consensus in” was achieved in round 3 (Kendall's *W* = 0.435, *p* < 0.001; all *CV* < 0.5). Round 1 recommended 10 categories with 73 indicators identified from 2,971 articles. After 3-round consultation, consensus was reached on 28 indicators focusing on the cardiovascular system (*n* = 4), oxygen transport system (*n* = 5), energy metabolism/metabolite level (*n* = 6), muscle/tissue damage level (*n* = 3), neurological function (*n* = 2), neuropsychological/psychological function (*n* = 3), endocrine function (*n* = 3), and exercise capacity (*n* = 2). Among these, 11 indicators were recommended as grade I: basic heart rate, heart-rate recovery time, heart rate variability, hemoglobin, blood lactic acid, urine protein, creatine kinase, reaction time, Borg Rating of Perceived Exertion Scale, testosterone/cortisol, and vertical jump height.

**Conclusion:**

This study developed a reliable foundation for the comprehensive evaluation of MIF among soldiers.

## Introduction

Exercise-induced fatigue (EIF) refers to a state in which the physiological function of the body cannot be kept at a certain level or the organism cannot maintain a predetermined exercise intensity (1). Military personnel, especially soldiers, regularly train or work with heavy loads in high intensity, often at risk of fatigue (2, 3). In China, the prevalence of fatigue among military personnel is significantly higher than the general population, especially the army and navy (4). Although fatigue among military personnel can be triggered by various factors, overtraining is the primary cause (5).

Military training-induced fatigue (MIF), a special type of EIF caused by military training, has become one of the obstructions affects the combat effectiveness of the army and led to the reduction of non-combat attrition (6). Therefore, management of MIF has strategic significance for maintaining combat capability.

However, the precise pathophysiological mechanism of MIF remains unclear. Some evidences show that the occurrence of MIF correlates with both physical and social-psychological factors (7, 8). Many researchers believe that MIF is brought out by complex mechanisms, which include central and peripheral nerve-muscle functional activities (9), cardiovascular and respiratory system functions (10), energy and substance metabolism (11), internal environment disorders (12), and fatigue control chain collapse (13). The uncertainty of the mechanism results in the uncertainty of MIF diagnosis and evaluation.

Many studies have researched EIF assessment among non-military personnel like athletes: they usually focus on one or two factors contributing to EIF, such as the type of exercise, location of muscle fatigue (14), immune and inflammatory alternation (15), physiological state (16) and other factors. Although there are various researches of EIF evaluation, up to now, the confirmed and widely-acknowledged tool of EIF assessment is only the Borg's Rating of Perceived Exertion Scale, a widely used psycho-physical tool to semi-quantitatively assess subjective perception of EIF (17), while objective specific indicator for the evaluation of EIF is still absent. Take blood lactic acid (LAC), one of the traditional discussed biomarkers of EIF, as an example. The “LAC hypothesis” for muscle fatigue, a kind of EIF, states that “accumulation of lactate or acidosis in working muscle causes inhibition of contractile processes, either directly or *via* metabolism, resulting in diminished exercise performance” (18). At present, LAC, as an indicator of energy metabolism, has been regarded as a biomarker of EIF in some intensive exercises such as swimming (19, 20) and cycling (21, 22). However, its cut-off value of swimming and cycling is still controversial. Besides, some studies also reported unchanged level of LAC among post-exercise persons who are evaluated as EIF by Borg's Rating of Perceived Exertion Scale (23). Therefore, though various indicators may be useful in evaluation EIF triggered by specific exercise type or among specific population, their efficacy in other exercise types like military training or special population like soldiers still remains unknown.

Military training or workload often covers a variety of modes of motion and various body parts. Thus, MIF of military personnel has difference between but also relationship with EIF among athletes of specific conventional sports, i.e., the measurement of EIF may be potential indicators of MIF but may also have its limitation and needs to be re-evaluated about its efficacy and applicability in MIF evaluation with specific factors considered. For example, hemoglobin (24), blood urea (25), and creatine kinase (26, 27) were reported as indicators of EIF among athletes. However, the study of Chen et al. (28) showed that among Chinese army special operation soldiers, levels of hemoglobin, blood urea, and creatine kinase had no significant difference between MIF and non-MIF soldiers, indicating the low-efficacy of hemoglobin, blood urea, and creatine kinase in the evaluation of MIF. Besides objective indicators, some psychological factors may also impact the degree of MIF. Previous study showed that physical inactivity has proven resistant to be trained and the decision to engage in exercise is based on psychological factors (29). For example, mood state, the main manifestation of psychological state is reported to have varied impact on person in and post exercise training (29). In America, the studies of Shannon et al. (30) observed depressive symptoms among soldiers with MIF. In China, though both positive and passive psychological state have been observed in new recruits during military training (31, 32). Our previous study manifested that basic combat training, a kind of common military training, could trigger MIF among soldiers with passive mood state while adding to the pleasure feeling among soldiers with positive mood state (33), indicating the potential efficacy of mood state in assessing MIF. What is more, social factors may also impact the psychological state of soldiers, which in turn, has influence on MIF (34).

In brief, though there are various studies explored the evaluation of EIF and MIF, at present, there is no research on the comprehensive evaluation of MIF based on physical and psychological indicators among military personnel such as soldiers.

Delphi methods or Delphi technique is a structured process used to anonymously collect opinions of individuals across diverse locations and areas of expertise to select indicators or develop healthcare framework, which could avoid domination of the consensus process by one or a few experts (35). Now, Delphi methods have been widely applicated for selecting healthcare quality indicators (36), involving sports medicine (37) and healthcare evaluation (38), among normal folks (39) and military population (40). A common Delphi consensus study is composed of four domains, i.e., questionnaire preparation, expert panel, progress of Delphi survey, and Delphi results assessment to achieve consensus (36). Generally, to suit for the questionnaire of different demands, the number of Delphi round and experts, and points-scoring principle of questionnaire items are varied among Delphi studies, therefore, modified Delphi methods (41) are widely applied.

On consideration the objectivity and authority of Delphi methods, and the fact that there is still no standard criterion for evaluating MIF. This study aimed to explore credible suggestions for evaluating MIF based on the current evidence and expert consensus *via* Delphi methods, so as to establish qualified criteria or a standard framework for the comprehensive evaluation of MIF. The results of this study could be a strong support for MIF prevention and cure among soldiers, contributing to the maintenance and improvement of combat effectiveness.

## Methods

### Study design

**Figure 1:**
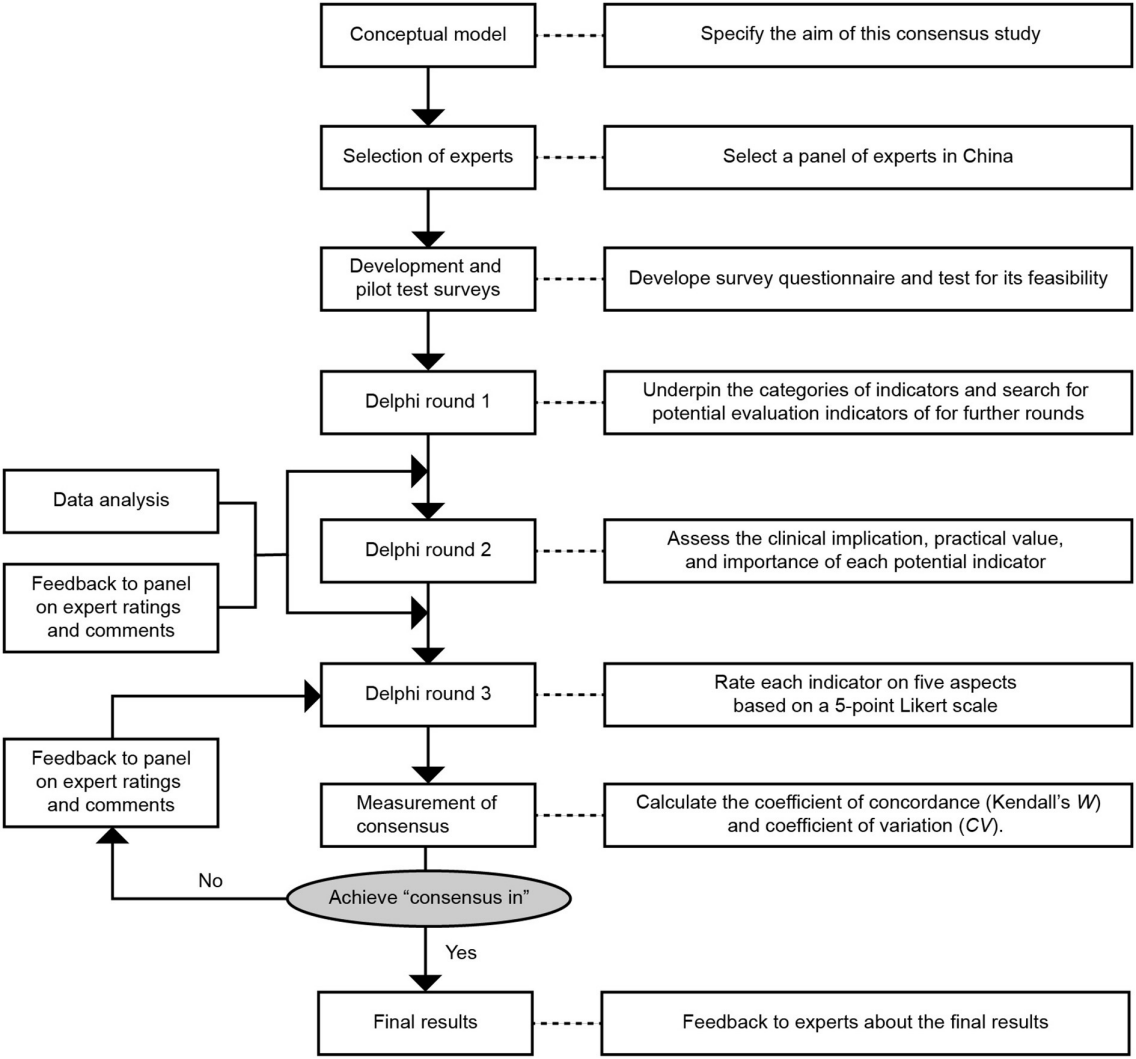
Process of this Delphi study.

This study was designed according to the reported Delphi method with modification (17), a method for achieving consensus among a panel of experts. In total, 3 rounds of the Delphi survey were conducted during January 2018 and January 2019 *via* letter. The study process is shown in Figure 1.

This study was approved by the Shanghai Changhai Hospital Ethnics Committee (No: 2018-048).

### Expert panel selection

A purposive sample of Chinese experts engaged in military medicine, sports and exercise medicine, rehabilitation medicine, kinesiology and exercise science, and medical laboratory science was selected for this Delphi study. The eligible experts had experience in their related profession more than 10 years, had a title of deputy equal to or higher than associate professor/associate chief physician/chief coach/associate researcher, and were available to complete all 3 rounds of surveys in Chinese before the required deadline.

All the experts included in this study were willing to participate and signed a written informed consent.

The related research of Delphi's expert consultation method (42) suggested that the preferable number of experts is 15–30: too few participants will limit the representation, and too many participants will result in low response and agreement rates and add to the complexity and cost. Therefore, the number of experts in this study is limited to this range.

### Delphi rounds

#### Delphi round 1

The first Delphi round aimed to underpin the categories of indicators and to search for potential evaluation indicators of MIF for further rounds.

In this round, first, experts who participated were asked to propose relative categories of indicators openly. Next, the researchers summarized the proposals into several categories and calculated the recommendation proportion of each category. Potential indicators were then searched *via* literature review. Database searches were performed in PubMed, Web of science, and four Chinese databases, including the China Biomedical Literature Database, the China National Knowledge Infrastructure, the Chongqing VIP Chinese Science and Technology Periodical Database, and the Wanfang databases. The search terms covered exercise-induced fatigue, fatigue, military training, soldier, and their synonyms (Supplementary material-Appendix 1). Studies published before January 31, 2018, were selected. Indicators used to evaluate the fatigue degrees of soldiers, athletes, and other participants after physical exercise were identified as potential.

#### Delphi round 2

Before this round, potential indicators classified into certain categories were introduced to participants as reference book A, which was written and edited by researchers according to the opinion of experts in round 1 and literature review. In this round, participants were asked to generally evaluate the clinical implication, practical value, and importance of each potential indicator, then vote for it. In this round, participants can recommend new indicators that were not involved in the reference book mentioned above and propose advice for revision of indicators, such as one indicator can be substituted by others. The recommendation proportion was calculated. Indicators with recommendation proportions ≥ 70% and new indicators recommended by participants would be included in round 3.

#### Delphi round 3

Before this round, the results of round 2 were reported to each participant as reference book B, which included the list of deleted indicators with reasons, the recommendation proportions of each indicator, and a literature summary of new indicators brought out in round 2. In this round, participants rated each indicator on five aspects based on a 5-point Likert scale (43). The five aspects are (1) the indicator is evidence-based (aspect 1), (2) the indicator is effective to evaluate MIF (aspect 2), (3) the indicator can prevent adverse events (aspect 3), (4) it is feasible to determine this indicator in the military (aspect 4), and (5) the indicator could be involved when measuring EIF among soldiers (aspect 5). Scores of 1 to 5 indicated disagree, slightly agree, agree, strongly agree, and completely agree, respectively.

#### Subject anonymity of Delphi round

Each Delphi round was conducted under the fully respect of subject anonymity as follows.

Before each Delphi round, researcher A sent our survey questionnaire and reference book to each expert *via* letter. Then within required time, experts should anonymously write their opinions, comments, and feedback in the survey questionnaire, and then send it back to researcher A. When researcher A collected back all experts' response, the anonymous paper version of response was sent to researcher B and C for data analysis.

Therefore, during this study, only researcher A knew the identity of experts, while all the opinions, comments, and feedback of experts were analyzed anonymously, and each expert get back other experts' viewpoint anonymously.

### Definition of consensus and recommendation set

According to the modified Delphi method (43), the consistency of participant opinions in this study was measured by the coefficient of concordance (Kendall's *W*) and coefficient of variation (*CV*). Kendall's *W* Test was conducted to calculate the value of Kendall's *W*, which is a number between 0 and 1, with higher values indicating better consistency of agreement (43). The *CV* of each aspect for each indicator was calculated as a standard deviation/mean of scores, with lower values indicating better consistency of agreement. “Consensus in” was achieved when Kendall's *W* of a round was between 0.2 and 0.5 and the *CV* of each aspect for an indicator was < 0.5 (44). If round 3 could not achieve “consensus in,” then more rounds would be conducted iteratively according to the process of round 3 (43), i.e., a round 4 or more should be conducted until whose Kendall's *W* is between 0.2 and 0.5 and *CV* < 0.5.

The indicators included in the recommendation set, namely, the recommended indicators, met the following criteria: the mean scores of aspects 1, 2, 3 and 5 are ≥ 3.0, and that of aspect 4 is ≥ 3.5. Additionally, we classified the recommend indicators into grade I (highly recommended) or grade II (recommended). The recommended indicators included in grade I met the following criteria: (1) the mean scores of aspects 1–5 are ≥ 3.5, and (2) the *CV* of aspect 5 is ≤ 0.25. The recommend indicators not included in grade I were classified into grade II.

### Quality assessment

The credibility of a Delphi study, which depends on the authority of the expert panel, is important to further research based on it. Thus, according to the Delphi method (43, 44), the quality of this study was assessed by the authority coefficient (*Aa*), which is the arithmetic mean value of the familiarity coefficient (*As*) and the coefficient of judgement basis (*Ai*). In this study, the value of *As* was calculated as the sum of familiarity scores of related disciplines (military medicine, sports and exercise medicine, rehabilitation medicine, kinesiology and exercise science, and medical laboratory science). Familiarity scores were reported by participants from 1.0 to 0 corresponding to the degree of familiarity as follows: very familiar (1.0 or 0.9), familiar (0.8 or 0.7), generally familiar (0.6, 0.5, or 0.4), unfamiliar (0.3 or 0.2) or very unfamiliar (0.1 or 0). The value of *Ai* was calculated as the sum of the judgement basis score, which was assigned based on theoretical analysis (0.30, 0.20, and 0.10), practical experience (0.50, 0.40, and 0.30), domestic and international references (0.10, 0.08, and 0.05) and intuitive judgement (0.10, 0.07, and 0.05) sequentially for large, medium, and small levels. The authority coefficient is positively correlated with the credibility. According to previous study, *Aa* > 0.7 indicates good credibility (43).

The levels of agreement between participants in the first, second, and third rounds were assessed with the intraclass correlation coefficient (ICC) as another evaluation of study reliability (45). The strength of reliability was defined as: very good (0.80 ≤ ICC < 1.00), good (0.60 ≤ ICC < 0.80), moderate (0.41 ≤ ICC < 0.60), fair (0.20 ≤ ICC < 0.40), and poor ( ≤ 0.20), with *p* < 0.05 (46).

### Data analysis

Response data from each round were transferred into electronic data by one researcher and checked by another researcher, then saved as Excel files (Microsoft office 2016). Data analysis was conducted using SPSS 21.0 (SPSS, Chicago, IL).

## Results

### Demographic characteristics of experts

Demographic characteristics of the Delphi panel are shown in Table 1. In total, 23 of the 40 invited experts agreed to participate in all the Delphi rounds. The mean age of the participants was 46.1 years (standard deviation [SD] = 5.4). The professional experience of the participants was 23.7 years on average (SD = 5.3). All 23 participants finished the Delphi survey in rounds 1, 2 and 3. The response rate of each round was 100.0%.

**Table 1:** Demographic characteristics of Delphi panel (*N* = 23).

**Characteristics**	***n***	**Percentage (%)**
**Gender**		
Male	19	82.6
Female	4	17.4
**Age (years)**		
<40	2	8.7
40–50	16	69.6
51–60	4	17.4
>60	1	4.3
**Experience in profession (years)**		
16–19	3	13.0
20–25	15	65.2
26–30	2	8.7
>30	3	13.0
**Title**		
Professor/chief physician/chief coach/researcher	14	60.9
Associate professor/associate chief physician/associate researcher	9	39.1
**Discipline**		
Military medicine	9	39.1
Sports and exercise medicine	4	17.4
Rehabilitation medicine	4	17.4
Kinesiology and exercise science	4	17.4
Medical laboratory science	2	8.7

### Delphi round 1

A total of 10 categories were put forward in this round. The recommendation proportions of categories varied from 34.8% to 100.0% (Table 2). Indicators of exercise capacity were recommended by all participants, followed by indicators of the cardiovascular system (91.3%), neuropsychological/psychological function (78.3%), and muscle/tissue damage (73.9%). The function of the respiratory, oxygen transport, neurological function, and energy metabolism/metabolite level were recommended by the majority to be important indicators of MIF degree (recommendation proportion > 60%, respectively). Although indicators showing endocrine and immune function were also recommended, the proportions were low.

**Table 2:** Recommended indicator categories.

**Indicator category**	**Recommendation proportion (%)**	**Number of indicators reported in the literature**
Exercise capacity	100.0	4
Cardiovascular system	91.3	6
Respiratory system	60.9	2
Oxygen transport system	60.9	2
Energy metabolism/metabolite level	69.6	18
Muscle/tissue damage	73.9	6
Neurological function	69.6	19
Neuropsychological/psychological function	78.3	1
Endocrine function	47.8	9
Immune function	34.8	6

**Figure 2:**
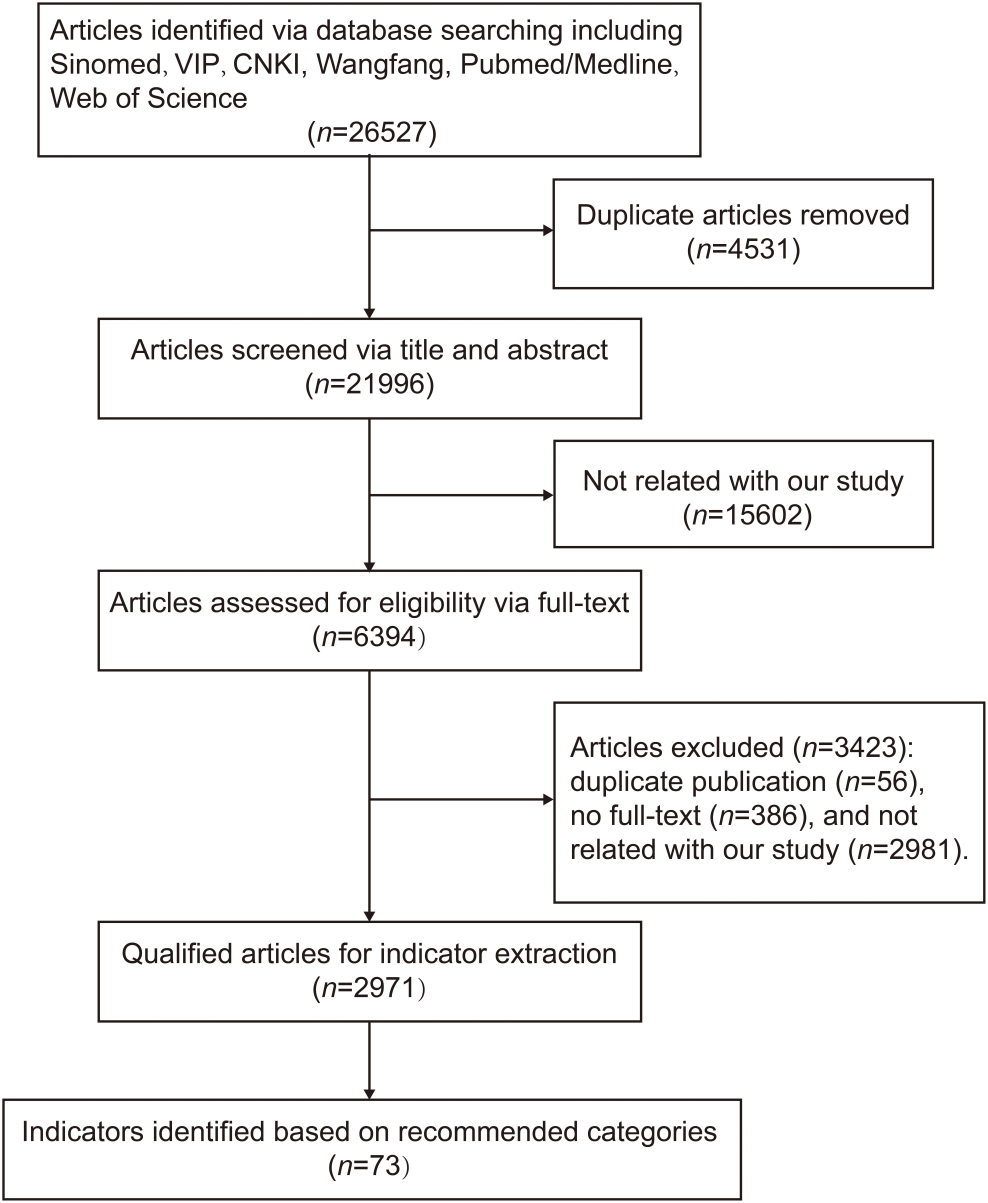
Process of literature review for potential indicators. CNKI, China National Knowledge Infrastructure; Sinomed, China Biomedical Literature Database; VIP, Chongqing VIP Chinese Science and Technology Periodical Database.

According to the categories recommended by experts, potential indicators were searched according to the database search strategy in Methods (section 2.3.1. Delphi round 1). The initial search retrieved 26,527 articles. After removing 4,531 duplications, screen *via* title and abstract (15602 removed), and assessed for eligibility *via* full text (3,423 removed), finally, 2,971 qualified articles were selected out. Among these 2,971 articles, a total of 73 indicators were identified according to the recommended 10 categories (Figure 2). Indicators evaluating the function of the nervous system and the energy metabolism/metabolite level constituted the majority, with a total proportion of 50.7% (*n* = 19 and 18, respectively). The number of indicators for each category is shown in Table 2. The number of articles per indicator is shown in Supplementary material-Appendix 2.

### Delphi round 2

All 73 indicators identified in round 1 were voted by the participants. The Kendall's *W* of this round was 0.174 (χ^2^: 287.835, *P* < 0.001). As shown in Table 3, the recommendation proportions varied from 30.4% to 100.0%. A total of 28 indicators had recommendation proportion ≥ 70% (Table 3), among which, the indicators Wingate test was recommended to be substituted by fatigue index and mean power, and muscle strength substituted by maximum voluntary contraction and twitch force (the experts' reasons were shown in Table 4). In addition, 14 new indicators were put forward by participants including 4 for oxygen transport system, 2 for neurological function, 3 for muscle/tissue oxidative damage and neuropsychological/psychological function respectively, and 1 for immune function and exercise capacity respectively (the indicators list and experts' reasons were shown in Table 5). Therefore, a total of 44 indicators were forwarded to round 3.

**Table 3:** Results of Delphi round 2.

**Category**	**Indicators forwarded to round 3 (*****n*** = **28)**			**Indicators excluded from round 3 (*****n*** = **45)**		
	**Indicator**	**RN**	**RP (%)**	**Indicator**	**RN**	**RP (%)**
Exercise capacity	Muscle strength	22	95.7	Countermovement jump height	16	69.6
	Wingate test	18	78.3	Harvard step index	14	60.9
Cardiovascular system	Electrocardiogram parameters	20	87.0	Blood pressure postural reflex	13	56.5
	Basic heart rate	23	100.0			
	Heart rate during exercise	20	87.0			
	Heart-rate recovery time	21	91.3			
	Heart rate variability	19	82.6			
Respiratory system	Vital capacity	20	87.0	Maximal inspiratory mouth pressure	11	47.8
Oxygen transport system	Hemoglobin	20	87.0			
	Red blood cell count	17	73.9			
Energy metabolism/metabolite level	Blood lactic acid	20	87.0	Lactate threshold	15	65.2
	Urine lactate	18	78.3	Lactate dehydrogenase	13	56.5
	Blood glucose	19	82.6	Pyruvate	11	47.8
	Blood ammonia	18	78.3	Alanine	12	52.2
	Blood urea	19	82.6	Glutamine	14	60.9
	Urine protein	21	91.3	Branched-chain amino acid	12	52.2
	Urine occult blood	17	73.9	Aromatic amino acid	11	47.8
				Creatinine	11	47.8
				Urobilinogen	13	56.5
				Nitric oxide	12	52.2
				Blood ketone	14	60.9
Muscle/tissue oxidative damage	Creatine kinase	20	87.0	Malondialdehyde	16	69.6
				Superoxide dismutase	15	65.2
				Catalase	11	47.8
				Glutathione peroxidase	10	43.5
				Total antioxidant capacity	11	47.8
Neurological function	Electromyogram parameters	20	87.0	γ-Aminobutyric acid (GABA)	16	69.6
	Tensiomyography parameters	17	73.9	Glutamate (Glu)	10	43.5
	Reaction time	23	100.0	Glu/GABA	11	47.8
	Threshold of skin space	18	78.3	5-Hydroxytryptamine (5-HT)	15	65.2
	Critical flicker frequency	17	73.9	Dopamine (DA)	12	52.2
				5-HT/DA	11	47.8
				Acetylcholine	14	60.9
				Noradrenaline	13	56.5
				Motor-evoked potentials (MEP)	11	47.8
				Cervicomedullary evoked potential (CMEP)	10	43.5
				MEP/CMEP	10	43.5
				Peripheral nerve stimulation-evoked M wave	10	43.5
				Electroencephalogram parameters	11	47.8
				Knee-jerk reflex threshold	9	39.1
Neuropsychological/psychological function	Borg Rating of Perceived Exertion Scale	22	95.7			
Endocrine function	Testosterone	17	73.9	Prolactin	12	52.2
	Cortisol	17	73.9	Growth hormone	14	60.9
	Testosterone/cortisol	19	82.6	Insulin	13	56.5
				Glucagon	10	43.5
				Antidiuretic hormone	10	43.5
				Prostaglandin	7	30.4
Immune function	White blood cell count	18	78.3	Immunoglobulin	16	69.6
				Interleukin-1	8	34.8
				Interleukin-6	14	60.9
				Interleukin-10	10	43.5
				Tumor necrosis factor-α	13	56.5

RN, recommendation number; RP, recommendation proportion.

**Table 4:** Expert's suggestions for two indicators in round 2.

**Original indicator**	**Indicator/index to substitute**	**Reason for recommendation**
Wingate test	(1) Fatigue index	This index is calculated as the amount of power drop during the Wingate test (47) and can be a quantitative measure of anaerobic fatigue.
	(2) Mean power (MP)	During the Wingate test, 20-s and 30- to 120-s MP generally reflect the energy supply capacities of the phosphagen system and glycolysis system, respectively (17). The averages of 10-, 30-, and 90-s MP can comprehensively reflect the energy supply capacity of the body, thereby reflecting the fatigue state of the body.
Muscle strength	(1) Maximum voluntary contraction	It is an indicator of muscle fatigue both centrally and peripherally (48), thus, it can reflect the comprehensive strength level of a target muscle group.
	(2) Twitch force (TF)	TF is the contraction force of a muscle under a single high-intensity electric stimulation; thus, it can measure the voluntary activation of muscle (9), which indicates the degree of muscle fatigue peripherally.

**Table 5:** New indicators put forward by experts in round 2.

**No**.	**Category**	**New indicators**	**Reason for recommendation**
1	Oxygen transport system	Maximum oxygen intake (VO_2_max)	VO_2_max is an important indicator of cardiopulmonary function and aerobic capacity. Although directly measuring the VO_2_max of soldiers is impractical, Cooper's 12-min run test can estimate VO_2_max by distance (m) (49). VO_2_max (mL/[kg·min]) = 22.351×distance (km) – 11.288 (50). Decrease of VO_2_max can indicate EIF.
2	Oxygen transport system	Serum transferrin	Physical exercise often reduces serum transferrin saturation, and anemic athletes with iron deficit have decreased physical performance (51). Thus, the level of serum transferrin should be considered to evaluate EIF.
3	Oxygen transport system	Serum ferritin	Serum ferritin could be used to evaluate iron status of athletes, while exercise-induced anemia caused by iron deficiency is closely related to subjective fatigue (52). Thus, the level of serum ferrin should be considered to evaluate EIF.
4	Oxygen transport system	Blood oxygen saturation	Exercise-induced arterial hypoxaemia is one factor contributing to muscle fatigue (53). Pulse oximetry monitoring is now feasible and portable. Thus, blood oxygen saturation monitoring can be used to evaluate EIF in real time.
5	Muscle/tissue oxidative damage	Serum myoglobin (Mb)	Mb, a sensitive indicator of muscle damage, decreases significantly during muscle fatigue (54). Compared to creatine kinase, Mb has a smaller molecular weight and thus is more sensitive for indicating muscle damage-related EIF.
6	Muscle/tissue oxidative damage	Urea 3-methylhistidine (3-MH)	3-MH is a sensitive index of myofibrillar protein degradation (55) and thus can indicate muscle damage.
7	Muscle/tissue oxidative damage	Serum bilirubin	Serum bilirubin increases significantly during exhausted exercises, such as marathons (56). Military training or action is always intensive and exhausting; thus, serum bilirubin can be an indicator of EIF among soldiers.
8	Neurological function	Transform growth factor-β (TGF-β)	TGF-β is closely related to central factors of EIF and tends to increase among the fatigue population (57).
9	Neurological function	Brain derived neurotrophic factor (BDNF)	BDNF is closely related to central factors of EIF (58). The decrease of BDNF could confine the maximal voluntary contraction and the central activation ratio (59).
10	Neuropsychological/psychological function	Delayed onset muscle soreness (DOMS)	DOMS is a main symptom of EIF and can be measured by Visual Analog Scale (60).
11	Neuropsychological/psychological function	Stroop test	The Stroop test assesses the executive function of the central nervous system (61) and thus could indicate the degree of central fatigue during EIF status.
12	Neuropsychological/psychological function	Profile of Mood State Questionnaire (POMS)	Psychological factors can contribute to the EIF of military personnel. POMS was invented by Australian psychologist Grove to assess mood state, and its Chinese version was translated and verified by professor Zhu with Cronbach α = 0.746 (62).
13	Immune function	C-reactive protein (CRP)	It is reported that inflammation contributes to the development of fatigue, and plasma CRP as an indicator was prospectively associated with new-onset fatigue (63), especially after high-intensity exercises (64).
14	Exercise capacity	Vertical jump height (VJH)	VJH is a simple test to evaluate exercise capacity. In China, VJH has been indexed in the Handbook of National Physical Fitness Determination Standards (65).

EIF, exercise-induced fatigue.

### Delphi round 3

**Figure 3:**
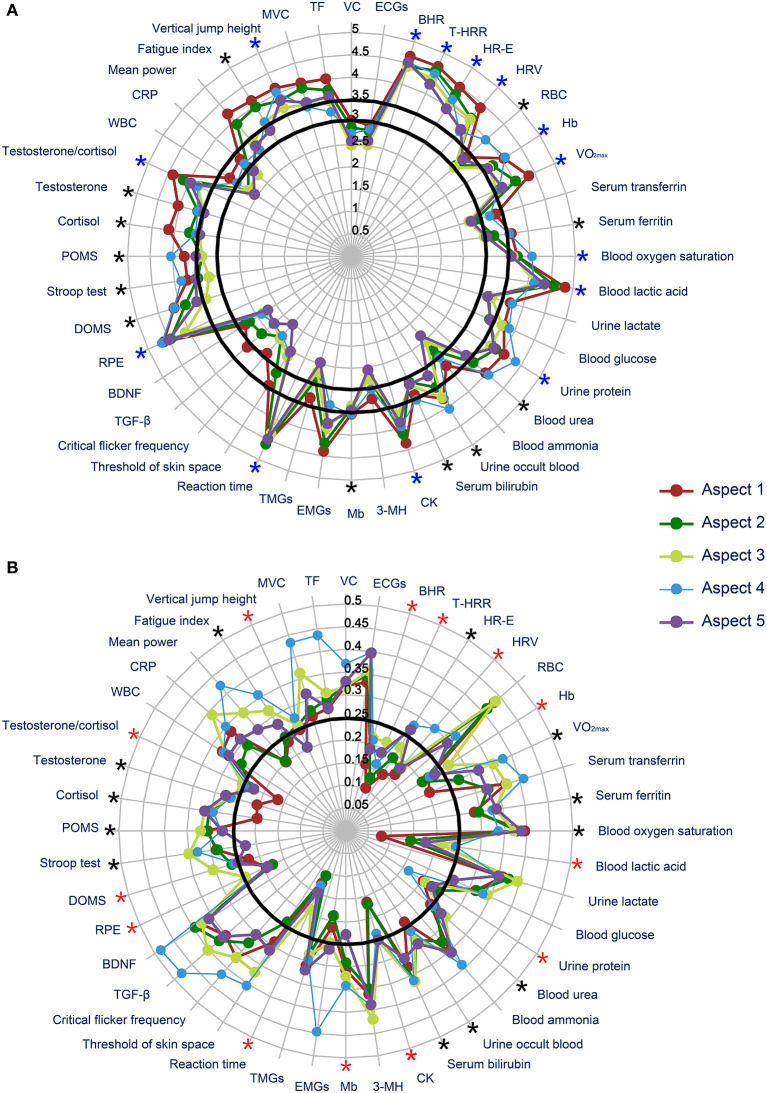
Results of round 3. **(A)** Mean score of each indicator in round 3; **(B)** coefficient of variation of each indicator in round 3. *in **black** color: indicators with mean scores of aspects 1, 2, 3 and 5 of ≥ 3.0 and that of aspect 4 of ≥ 3.5; *in **blue** color: indicators with mean scores of aspects 1–5 of ≥ 3.5; *in **red** color: indicators with *CVs* of aspect 5 of ≤ 0.25. VC, vital capacity; ECGs, electrocardiogram parameters; BHR, basic heart rate; T-HRR, heart-rate recovery time; HR-E, heart rate during exercise; HRV, heart rate variability; RBC, red blood cell count; Hb, hemoglobin; VO_2max_, maximum oxygen intake; CK, creatine kinase; 3-MH, urea 3-methylhistidine; Mb, serum myoglobin; EMGs, electromyogram parameters; TMGs, tensiomyography parameters; TGF-β, transform growth factor-β; BDNF, brain-derived neurotrophic factor; RPE, Borg's Rating of Perceived Exertion Scale; DOMS, delayed onset muscle soreness; POMS, Profile of Mood State Questionnaire; WBC, white blood cell count; CRP, C-reactive protein; MVC, maximum voluntary contraction; TF, twitch force.

The Kendall's *W* of round 3 is 0.435 (χ^2^: 430.607, *P* < 0.001). The mean score and *CV* of each aspect of the individual indicators fluctuated between 2.00 and 4.83 (Figure 3A) and between 0.080 and 0.484, respectively (Figure 3B). The value of Kendall's *W* (> 0.2) and *CV* (all < 0.5) indicated this round achieved “consensus in.” As shown in Figure 3A, 28 indicators meet the inclusion criteria of the mean score for the recommendation set, among which 14 meet the criteria for grade I (highly recommended). As shown in Figure 3B, only 13 indicators meet the inclusion criteria of the *CV* for grade I (highly recommended), among which 2 indicators (serum myoglobin and delayed-onset muscle soreness) did not meet the mean score criterion. Thus, a total of 28 indicators were included, with 11 classified as grade I and 17 as grade II (Figure 3 and Table 6). As shown in Table 6, the categories covered cardiovascular system (*n* = 4), oxygen transport system (*n* = 5), energy metabolism/metabolite level (*n* = 6), muscle/tissue damage (*n* = 3), neurological function (*n* = 2), neuropsychological/psychological function (*n* = 3), endocrine function (*n* = 3), and exercise capacity (*n* = 2).

**Table 6:** Recommend indicators.

**Category**	**Recommend indicators**	
	**Grade I (*n* = 11)**	**Grade II (*n* = 17)**
Cardiovascular system (*n* = 4)	Basic heart rate, heart-rate recovery time, heart rate variability	Heart rate during exercise
Oxygen transport system (*n* = 5)	Hemoglobin	Red blood cell count, maximum oxygen intake, serum ferritin, blood oxygen saturation
Energy metabolism/metabolite level (*n* = 6)	Blood lactic acid, urine protein	Urine lactate, blood glucose, blood urea, urine occult blood
Muscle/tissue damage (*n* = 3)	Creatine kinase	Serum myoglobin, serum bilirubin
Neurological function (*n* = 2)	Reaction time	Stroop test
Neuropsychological/psychological function (*n* = 3)	Borg Rating of Perceived Exertion Scale	Delayed onset muscle soreness (DOMS), Profile of Mood State Questionnaire (POMS)
Endocrine function (*n* = 3)	Testosterone/cortisol	Testosterone, cortisol
Exercise capacity (*n* = 2)	Vertical jump height	Fatigue index

### Quality assessment

The mean familiarity coefficient and judgement coefficient were 0.64 and 0.83, respectively; thus, the mean authority coefficient was 0.733 (> 0.7). The authority of experts was good, and the results of the 3-round consultation were credible.

The ICC for the first, second, and third round were 0.821 (95% confidential interval [CI]: 0.607–0.947, *p* < 0.001), 0.787 (95% CI: 0.710–0.852, *p* < 0.001), and 0.932 (95% CI: 0.899–0.958, *p* < 0.001), which all meet the “very good (0.80 ≤ ICC < 1.00) or good (0.60 ≤ ICC < 0.80)” criteria of reliability, further illustrating the good reliability of this Delphi study.

## Discussion

### Summary of findings

The Delphi method is a way to solicit expert opinions to gain consensus by iterative stages of anonymous responses (66). The goal of this method is to reduce the range of responses to gain expert consensus quantitatively and qualitatively, which is often seen as more credible than conjecture or individual opinion (42).

A total of 23 experts participated all Delphi rounds, whose average professional experience year was 23.7 years. The mean authority coefficient of experts was 0.733, indicating good authority of participants. The majority of experts are male (19/23, 82.6%) and aged between 40 and 50 years. As previous comparison study exploring the potential confounding factor of Delphi consensus reported (67), no significant difference was observed between men and women experts when giving their opinion, but the authority of experts impacts their opinion significantly. In our study, to achieve a high authority of experts, we set the qualification criteria of experts as “has experience in their related profession more than 10 years, had a title of deputy equal to or higher than associate professor/associate chief physician/chief coach/associate researcher,” which results to the experts aged between 40 and 50 years.

In round 2, 14 new indicators were put forward by participants to evaluate MIF *via* oxygen transport function (4/14, 28.57%), muscle/tissue oxidative damage levels (3/14, 21.43%), neurological and psycho-behavioral functions (5/14, 35.71%), immune system function (1/14, 7.14%), and exercise capacity (1/14, 7.14%). The results showed that, on the one hand, in the previous literature research (Figure 2), the research group still had some shortcomings and failed to comprehensively identify potential indicators of MIF; on the other hand, the factors involved in MIF may be much larger than we surmised: both the physical and psychological status of soldiers should be considered when evaluating MIF. As previous study on the influence of the literature searches reported (68), the amount of information available (i.e., keywords, bibliographics, abstracts) and the cognitive characteristics of the searcher could both lead to different search outcomes. In our study, literature search aimed to identify potential indicators of MIF. The initial search retrieved 26,527 articles. When removing duplications (*n* = 4,531), there are still a huge number of articles should be screened for checking their eligibility. Therefore, careless omission or exclusion of literature could not be avoidable, which partially leads to the gap between literature search and experts' recommendation. While, for physical and psychological status, our research strategy did not pinpoint on keywords “physical” or “psychological,” which may also partially lead to leak detection. The above results acknowledged the value of extensive expert consultation and multidisciplinary knowledge exchange in the exploration of MIF indicators and comprehensive evaluation.

After 3 rounds of consultation, a total of 28 recommended indicators (grades I and II) were screened out. Most of these indicators were involved in evaluating MIF *via* cardiopulmonary and oxygen transport system function (9/28, 32.14%) and energy metabolism/metabolite level (6/28, 21.43%). These results were consistent with the current “wear-out doctrine” and “blockage doctrine” of the mechanism of MIF: that is, the consumption of a large amount of energy substances and accumulation of metabolites during physical exercise/training leads to a decline in the function capacity of tissues, muscles, and organs, ultimately resulting in fatigue (17). The function of the cardiopulmonary and oxygen transport systems is the basis of substance metabolism of energy supply during the exercise process, and the level of material energy metabolism can further regulate the function of the cardiopulmonary and oxygen transport systems (17). Therefore, these two systems have an important role in maintaining the movement of the human body, and thus, should be considered in the evaluation of MIF. Besides cardiopulmonary and oxygen transport system function, and energy metabolism/metabolite level, this Delphi consultation also recommended indicators about muscle/tissue damage, neurological function, neuropsychological/psychological function, endocrine function, and exercise capacity. These results were consistent with previous study about the relationship between exercise and fatigue: physical exercise affects the equilibrium of the internal environment of various physical systems which in turn create sensations of fatigue and exhaustion in the mind of the exercising subject (69). Muscle/tissue damage indicators like creatine kinase are related to the injury of muscle cell triggered by forced myotasis during exercise/training (26, 27), and the injured muscle cells will decrease the exercise capacity of a person, therefore, leading to fatigue. The change of neurological function is one manifestation of fatigue originating from central nervous system. Some invasive indicators such as motor-evoked potentials of transcranial magnetic stimulation (70) were reported before. However, invasive examination has difficulty in the application among soldiers. In our study, reaction time (71) and Stroop test (61) recommended after three-round consensus could avoid the defects of application, further acknowledged the value of extensive expert consultation among indicator selection. For indicators on endocrine function, testosterone (72) and cortisol (73) were reported to be related to the degree of EIF among male athletes. Although their application in female athletes remained unknown, for military populations, gender limitation (the majority are men) on the contrary adds to their feasibility in assessing MIF. Finally, though decreasing of exercise capacity is the most direct symptom of MIF, how to conduct exercise capacity test without adding to the degree of fatigue is an important issue. The grade I indicator recommended by our study is vertical jump height, a simple test that will almost not deteriorate exercise capacity (65), could be a good choice for MIF evaluation.

The results of response rate, quality assessment, Kendall's *W* and *CV* showed satisfied attendance of experts (100%), good credibility of this study (authority coefficient > 0.7), and consistency of experts' opinions (Kendall's *W* = 0.435, all *CV* < 0.5 in round 3), indicating this Delphi-consensus study made a reliable recommendation about the indicators that may be potentially useful in the comprehensive evaluation of MIF among soldiers. In China, this is the first MIF-evaluation study focusing on military personnel; thus, it may benefit further research on the management of MIF among soldiers.

### Strengths and limitations

This study initially clarified the value of comprehensive evaluation in the diagnosis and assessment of MIF. This study better followed the principle of Delphi consensus, and therefore, is a good reference for the management of MIF. But anyway, there do be some limitations. Firstly, this consensus study based on the previous literature of fatigue evaluation and the experience of expertise, therefore, the efficacy of recommended indicators in evaluation MIF needs further study to confirm. Secondly, this consensus study recommends to evaluate MIF comprehensively based on eight aspects of physical systems/capacity, but further comprehensive frame of these aspects and specific combination of indicators needs more sectional and cohort studies to support.

### Implications for policy, practice and research

In China, this is the first MIF-evaluation study focusing on military personnel; thus, it could benefit further research on the management of MIF among soldiers. This consensus study indicates the necessary to evaluate MIF in a comprehensive way. Further researches of MIF will focus on constructing a comprehensive evaluation framework for MIF diagnosis and management.

## Conclusion

This 3-round Delphi consensus study developed a reliable foundation about the comprehensive evaluation of MIF among soldiers. Although more clinical studies should be performed to confirm the diagnostic efficacy of each potential indicator among soldiers with MIF, the recommendation of indicators in grades I and II provided a more detailed and operable reference for evaluating MIF in the Chinese military. Also, since the military population includes female soldiers, gender is an important factor that must be considered when evaluate MIF with potential indicators like neurophysiological/psychological function, endocrine function, etc.

## Data availability statement

The original contributions presented in the study are included in the article/Supplementary material, further inquiries can be directed to the corresponding author/s.

## Author contributions

WG and C-qL put forward the conception and design of the study. YR, S-jS, MW, and NH collected the original data. S-jS, Z-fY, and YR analyzed and interpreted the data. Z-fY, WG, and C-qL revised it critically. All authors drafted the article and approved the final version to be submitted.

## Funding

This study was supported by National Science and Technology Major Project (No: 2018ZX09J18110-003-002), Military Special Program to Cultivate and Improve TCM Service Capability (2021ZY002), and Military Health Care Special Scientific Research Project (22BJZ16) but not in data analysis and preparation of the article, writing of the report; and in the decision to submit the article for publication.

## Conflict of interest

The authors declare that the research was conducted in the absence of any commercial or financial relationships that could be construed as a potential conflict of interest.

## Publisher's note

All claims expressed in this article are solely those of the authors and do not necessarily represent those of their affiliated organizations, or those of the publisher, the editors and the reviewers. Any product that may be evaluated in this article, or claim that may be made by its manufacturer, is not guaranteed or endorsed by the publisher.
