# Supplementary material for: Comprehensive evaluation of military training-induced fatigue among soldiers in China: A Delphi consensus study
Source: Front Public Health. 2022 Nov 29;10:1004910. doi: 10.3389/fpubh.2022.1004910 (PMC9745162; doi:10.3389/fpubh.2022.1004910)
Supplement: Supplementary file 1 [file Data_Sheet_1.docx]

**Appendix 1**

**Search strategy for literature review in round 1 Delphi study**

**1. PubMed**

((((military) AND fatigue)) OR exercise-induced fatigue) OR ((training) AND fatigue AND Humans [Mesh])

**2. Web of science**

# 1 TOPIC: (military) OR TITLE: (military)

# 2 TOPIC: (training) OR TITLE: (training)

# 3 TOPIC: (fatigue) OR TITLE: (fatigue)

# 4 (# 1 OR # 2) AND # 3

**3. China Biomedical Literature Database**

| Original Search Strategy | Translation in English |
| --- | --- |
| # 1 "军事"[常用字段] AND ("疲劳"[常用字段] OR "倦怠"[常用字段] OR "疲劳"[主题词]) | # 1 "Military"[all field] AND ("Fatigue"[all field] OR "tired"[all field] OR "Fatigue"[subject terms]) |
| # 2 "训练"[常用字段] AND ("疲劳"[常用字段] OR "倦怠"[常用字段] OR "疲劳"[主题词]) | # 2 "Training"[all field] AND ("Fatigue"[all field] OR "tired"[all field] OR "Fatigue"[subject terms]) |
| # 3 "运动性疲劳"[常用字段] AND ("运动性疲劳"[主题词]) | # 3 "Exercise-induced fatigue"[all field] AND ("Exercise-induced fatigue"[ subject terms]) |
| # 4 # 1 OR # 2 OR # 3 | # 4 # 1 OR # 2 OR # 3 |

**4. China National Knowledge Infrastructure**

| Original Search Strategy | Translation in English |
| --- | --- |
| # 1 "军事"[常用字段] AND ("疲劳"[常用字段] OR "倦怠"[常用字段] OR "疲劳"[主题词]) | # 1 "Military"[all field] AND ("Fatigue"[all field] OR "tired"[all field] OR "Fatigue"[subject terms]) |
| # 2 "训练"[常用字段] AND ("疲劳"[常用字段] OR "倦怠"[常用字段] OR "疲劳"[主题词]) | # 2 "Training"[all field] AND ("Fatigue"[all field] OR "tired"[all field] OR "Fatigue"[subject terms]) |
| # 3 "运动性疲劳"[常用字段] AND ("运动性疲劳"[主题词]) | # 3 "Exercise-induced fatigue"[all field] AND ("Exercise-induced fatigue"[ subject terms]) |
| # 4 # 1 OR # 2 OR # 3 | # 4 # 1 OR # 2 OR # 3 |

**5. Chongqing VIP Chinese Science and Technology Periodical Database**

| Original Search Strategy | Translation in English |
| --- | --- |
| (M=(训练 AND 疲劳) OR (M=(军事 AND 疲劳)) OR (M=运动性疲劳) | (M=(Training AND Fatigue) OR (M=(Military AND Fatigue)) OR (M=Exercise-induced fatigue) |

M: Title or keywords

**6. Wanfang databases**

| Original Search Strategy | Translation in English |
| --- | --- |
| 主题:(“训练”)*主题:(“疲劳”)OR主题:(“军事”)*主题:(“疲劳”)OR主题:(“运动性疲劳”) | Subject:(“Training”)*Subject:(“Fatigue”) OR Subject:(“Military”)*Subject:(“Fatigue”) OR Subject:(“Exercise-induced fatigue”) |
