# Supplementary material for: Comprehensive evaluation of military training-induced fatigue among soldiers in China: A Delphi consensus study
Source: Front Public Health. 2022 Nov 29;10:1004910. doi: 10.3389/fpubh.2022.1004910 (PMC9745162; doi:10.3389/fpubh.2022.1004910)
Supplement: Supplementary file 2 [file Table_1.docx]

**Appendix 2**

**Table S.** Number of articles per potential indicator in Delphi round 1.

| Indicator category | Indicator reported in literature | Number of relative articles* |
| --- | --- | --- |
| Exercise capacity | Muscle strength | 335 |
|  | Wingate test | 68 |
|  | Countermovement jump height | 53 |
|  | Harvard step index | 44 |
| Cardiovascular system | Electrocardiogram parameters | 43 |
|  | Basic heart rate | 78 |
|  | Heart rate during exercise | 92 |
|  | Heart-rate recovery time | 42 |
|  | Heart rate variability | 44 |
|  | Blood pressure postural reflex | 6 |
| Respiratory system | Vital capacity | 45 |
|  | Maximal inspiratory mouth pressure | 6 |
| Oxygen transport system | Haemoglobin | 71 |
|  | Red blood cell count | 49 |
| Energy metabolism/metabolite level | Blood lactic acid | 667 |
|  | Urine lactate | 76 |
|  | Lactate threshold | 44 |
|  | Blood ammonia | 9 |
|  | Blood urea | 99 |
|  | Blood glucose | 93 |
|  | Urine occult blood | 33 |
|  | Urine protein | 31 |
|  | Lactate dehydrogenase | 62 |
|  | Pyruvate | 45 |
|  | Alanine | 65 |
|  | Glutamine | 36 |
|  | Branched-chain amino acid | 42 |
|  | Aromatic amino acid | 6 |
|  | Creatinine | 39 |
|  | Urobilinogen | 5 |
|  | Nitric oxide | 85 |
|  | Blood ketone | 6 |
| Muscle/tissue damage | Creatine kinase | 218 |
|  | Malondialdehyde | 48 |
|  | Superoxide dismutase | 73 |
|  | Catalase | 42 |
|  | Glutathione peroxidase | 47 |
|  | Total antioxidant capacity | 35 |
| Immune function | White blood cell count | 24 |
|  | Immunoglobulin | 19 |
|  | Interleukin-1 | 42 |
|  | Interleukin-6 | 70 |
|  | Interleukin-10 | 12 |
|  | Tumour necrosis factor-α | 2 |
| Neurological function | Electromyogram parameters | 64 |
|  | Tensiomyography parameters | 9 |
|  | Reaction time | 62 |
|  | Threshold of skin space | 6 |
|  | Critical flicker frequency | 5 |
|  | γ-Aminobutyric acid (GABA) | 34 |
|  | Glutamate (Glu) | 42 |
|  | Glu/GABA | 15 |
|  | 5-Hydroxytryptamine (5-HT) | 52 |
|  | Dopamine (DA) | 77 |
|  | 5-HT/DA | 43 |
|  | Acetylcholine | 39 |
|  | Noradrenaline | 39 |
|  | Motor-evoked potentials (MEP) | 11 |
|  | Cervicomedullary evoked potential (CMEP) | 15 |
|  | MEP/CMEP | 4 |
|  | Peripheral nerve stimulation-evoked M wave | 14 |
|  | Electroencephalogram parameters | 11 |
|  | Knee-jerk reflex threshold | 2 |
| Neuropsychological/psychological function | Borg Rating of Perceived Exertion Scale | 231 |
| Endocrine function | Testosterone | 62 |
|  | Cortisol | 115 |
|  | Testosterone/cortisol | 38 |
|  | Prolactin | 27 |
|  | Growth hormone | 36 |
|  | Insulin | 135 |
|  | Glucagon | 15 |
|  | Antidiuretic hormone | 2 |
|  | Prostaglandin | 29 |

*One article could include more than one indicator.
